# Multidrug-Resistant and Genetic Characterization of Extended-Spectrum Beta-Lactamase-Producing *E. coli* Recovered from Chickens and Humans in Egypt

**DOI:** 10.3390/ani12030346

**Published:** 2022-01-31

**Authors:** Heba Badr, Reem M. Reda, Naglaa M. Hagag, Essam Kamel, Sara M. Elnomrosy, Amal I. Mansour, Momtaz A. Shahein, Samah F. Ali, Hala R. Ali

**Affiliations:** 1 Reference Laboratory for Veterinary Quality Control on Poultry Production, Animal Health Research Institute, Agriculture Research Center (ARC), Giza 12618, Egypt; drheba_badr@yahoo.com (H.B.); reda_reem@yahoo.com (R.M.R.); naglaahagagahri@gmail.com (N.M.H.); 2 Department of chemistry, Animal Health Research Institute, Agriculture Research Center (ARC), Giza 12618, Egypt; essamkamel1@yahoo.com; 3 Genome Research Unit, Animal Health Research Institute, Agriculture Research Center (ARC), Giza 12618, Egypt; dr_sara_vet_2007@yahoo.com; 4 Bacteriology, Food and Agriculture Organization of the United Nations, Giza 2223, Egypt; Amal.Mansour@fao.org; 5 Department of Virology Research, Animal Health Research Institute, Agriculture Research Center (ARC), Giza 12618, Egypt; momtaz.shahein@yahoo.com; 6 Bacteriology Department, Animal Health Research Institute (AHRI), Agriculture Research Center (ARC), Giza 12618, Egypt; samah_hefny2004@yahoo.com

**Keywords:** antibiotic resistance, *E. coli*, ESBL, poultry, human

## Abstract

**Simple Summary:**

Feeding food chain animals with sub-therapeutic doses for prophylaxis or for growth-promoting purposes has led to the emergence of resistant bugs such as ESBL-*E. coli*. Infections caused by these superbugs are tremendously associated with treatment failures and high morbidity/mortality rates. Scarce information is currently available on the relation between the incidence of ESBL-*E. coli* in human and food chain animals in Egypt. The current study analyzed chicken and human fecal samples for isolation and characterization of ESBL-producing *E. coli* followed by sequencing the isolates. Significant similarities were detected between human and chicken isolates, indicating the possibility of zoonotic transmission. In conclusion, the study encouraged managing the use of antibiotics in veterinary field, to reduce the selection and spread of life-threating bugs to humans.

**Abstract:**

Colonization of food chain animals such as chickens with extended-spectrum β-lactamases (ESBL) poses a major health threat to human. The current study aimed to determine the phenotypic and genotypic relationship between ESBL-producing *E. coli* from diseased human and chickens in Egypt. A total of 56 out of 120 chicken farms (46.7%) and 9 human samples (100%) were phenotypically and genotypically identified with at least one ESBL-phenotype/gene. Chicken isolates showed a high proportion of beta lactamase from CTX-M group 9 > TEM > PER families, followed by CTX-M group 1 > SHV > GES > OXA group10 > VEB > OXA group2 families, while human isolates only contained the CTX-M family. A high incidence of ESBL genes from the CTX-M family was recognized in both human and chicken isolates. Furthermore, nucleotide identity showed high similarity between chicken and human isolates. In conclusion, the current study traced phenotypes and genotypes of ESBL-producing *E. coli* from chickens and human samples in Egypt, reporting degrees of similarity that suggest potential zoonotic transmission. Our data highlighted the significant importance of chicken as a major food source not only in Egypt but all over the world in the spreading of ESBL-producing *E. coli* to human.

## 1. Introduction

*Escherichia coli* is a member of the family *Enterobacteriaceae* which causes enteric and extraintestinal infections in both animals and humans [1]. The resistance of *Enterobacteriacea* to third- and fourth-generation cephalosporins via the production of extended-spectrum β-lactamases (ESBLs) has raised since the 2000s, limiting therapeutic options against these infections [2]. The potential zoonotic importance of *E. coli* either from animals or food derived from animals has been previously documented. In the last two decades, *E. coli*-producing ESBLs have been reported widely in animals as well as humans possessing a serious public-health threat [3,4].

Due to the global spread of antibiotic-resistance bacteria, the World Health Organization (WHO), Food and Agriculture Organization of the United Nation (FAO), and World Organization for Animal Health (OIE) have coined the “One Health” approach, which includes collaborative efforts from the environment, animal, and human health authorities to limit the spread of this phenomenon [5,6]. Various scenarios have shown that close contact between humans and infected livestock contributes to spreading of resistant bacteria including ESBL-producing *E. coli.* Since human and animals share the same environment, they potentially share resistant bacteria and resistant genes [7]. It has been shown that poultry and poultry by-products act as a potential source of antibiotic-resistant bacteria, including ESBL-producing *E. coli,* to humans [8].

Nine distinct structural and evolutionary families of ESBL variants were reported based on amino acid sequence comparisons, such as TEM, SHV, CTX-M, PER, VEB, GES, BES, TLA, and OXA. The major ESBL variants are TEM, SHV, CTX-M, and OXA. The *bla_CTX−M_* variant is rapidly spreading and widely reported in *E. coli* around the globe [9]. African countries have reported a significant incidence of ESBL-producing *E. coli* among humans and animals. ESBL-*E. coli* with *bla_CTX-M_*, *bla_SHV_*, and *bla_TEM_* genes were previously detected in 20.1% chicken markets in Zambia [10]. In Ghana, ESBL-producing *E. coli* were found in humans and broilers chickens harboring *bla_CTX-M_* family [11]. Moreover, a number of studies suggested that chicken meat and meat products might be a potential source of ESBL-bacteria transmission to humans in Africa [11,12]. Comparable analysis conducted in central Europe demonstrated genetic similarity in ESBL-producing *E. coli* from Mongolian migratory birds and clinical isolate from hospitalized human in Europe [13]. Additionally, number of studies have shown frequent colonization of poultry with ESBL-producing *E. coli* [14,15], which puts humans in contact with and consumers at risk of acquiring ESBL infections. In support, an association has been noticed between the colonization of chicken retail meat with ESBL-producing *E. coli* and a high incidence of ESBL infections in hospitalized patients in the Netherlands [16].

In Egypt, ESBL-producing *E. coli* was previously reported in chickens, chicken meat [17,18], and humans [3], which highlights the importance of continuous monitoring ESBL-producing *E. coli* in both animals and humans. Hence, this study was aimed to investigate the phenotypic and genotypic characteristics of ESBL-producing *E. coli* samples obtained from diseased chickens and compared with samples retrieved from human cases in Egypt in the period between 2019 and 2020.

## 2. Materials and Methods

### 2.1. Sample Collection

A total of 120 diseased broiler chicken farms (five chickens from each farm and the age of birds varied from 7 to 35 days) suffered from ruffled feathers, depression, and loss of appetite. Samples were collected from different geographical locations in the northern part of Egypt (Dakahlia, Giza, and Sharqia Governorates) between September 2019 to December 2020. Birds were transported to the Reference Laboratory for Veterinary Quality Control on poultry production and further subjected to post mortem examination under septic conditions. Samples were obtained from internal organs (liver, lung, spleen, and heart) from birds showing colisepticemia, air sacculitis, perihepatitis, and pericarditis and pooled together for bacterial screening and isolation. In parallel, nine stool samples were collected from diseased humans from Qena. All samples collection procedures were legally approved by the Committee of Ethics at the Animal Health Research Institute, Egypt, under protocol number (AHRI-42429).

### 2.2. Isolation and Identification 

*E. coli* was isolated and identified as described elsewhere [19]. Briefly, samples were incubated aerobically into buffer peptone water at 37 °C for 24 h. A loopful from each incubated sample was streaked onto MacConkey’s agar (Oxoid, Manchester, UK) and Eosin Methylene Blue agar (Lioflichem, Roseto degli Abruzzi, Italy) plates were then incubated at 37 °C for 24 h. The suspected colonies were 1–2 mm diameter, and appeared as a hot-pink color colony on MacConkey and metallic sheen colonies on Eosin Methylene Blue agar. Suspected *E. coli* colonies were subjected for further biochemical examination (indole test, methyl red, voges Proskauer “VP”, citrate utilization, oxidase test, and Triple Sugar Iron “TSI”). Furthermore, serotyping of isolated *E. coli* was performed using Somatic (O) antigens and antiserum according to the kit instruction of (DENKA SEIKEN Co., Tokyo, Japan).

### 2.3. Antimicrobial Susceptibility Pattern and ESBL Screening of the Isolated E. coli

#### 2.3.1. Antimicrobial Sensitivity Test (AST)

AST was performed for all isolates by the disc diffusion test as previously described [20] against 14 antibiotics (Oxoid, Basingstoke, UK). Furthermore, initial ESBL screening was carried out for all isolates by disc diffusion method based on various cephalosporins according to the Clinical and Laboratory Standards Institute (CLSI) standard [21]. Isolates with an inhibition zone size of ≤22 mm with ceftazidime (30 μg), ≤25 mm with ceftriaxone (30 μg), ≤27 mm with cefotaxime (30 μg), and ≤27 mm with Aztreonam (30 μg) were identified as potential ESBL producers.

#### 2.3.2. Double Disc Synergy Test (DDST)

To confirm ESBL production, DDST was performed as described elsewhere [22]. Briefly, Amoxicillin-clavulanic acid (AMC, 30 μg) applied with a distance 20 mm center-to-center to that of each antibiotic disc (30-μg) of third-generation cephalosporin (Cefotaxime and Ceftriaxone) and fourth-generation cephalosporins (Ceftazidime and Cefepime) on Mueller-Hinton Agar (MHA) plates. Clear extension of the edge of the inhibition zone of cephalosporin toward the AMC disc was interpreted as positive for ESBL production. 

HiCrome ESBL agar (Himedia®, Mumbai, India). This test was also used for identification of ESBL *E. coli*. It is a rapid test, as it gives a result in approximately 24 h. ESBL *E. coli* producers show pink or purple colonies.

### 2.4. Genotypic Characterizations of ESBL

All isolates were initially tested using polymerase chain reaction (PCR) specific for the presence of ESBL genes: *bla_TEM_*, *bla_SHV_*, *bla_CTX-M_*, *bla_OXA_*, *bla_GES_*, *bla_VEB_*, and *bla_PER_*, as described previously [9,23]. Briefly, DNA extraction was performed using QIAamp DNA extraction Kit (Qiagen, Hilden, Germany) according to the manufacturer’s instructions. The extracted DNA was further tested using gene-specific PCR assays using COSMO PCR RED Master Mix (Willowfort, Birmingham, UK). Amplification was performed using Bio-Rad thermal cycler and consisted of an initial denaturation (1 cycle) for 10 min at 95 °C followed by 35 cycles of denaturation: 96 °C/3 s, annealing: 58–67 °C/10 s, extension: 68 °C/15 s; and a final extension cycle for 15 s at 72 °C. Primers used with different annealing temperature are detailed in Table 1.

Six housekeeping genes *adk, fumC, gyrB, icd, mdh,* and *purA* were amplified for three chicken and two human isolates using primers previously described by [24]. Amplification was carried out in a 50 µL reaction containing 25 µL of COSMO PCR RED Master Mix (Cat. No. 1020300X), 2.5 µL of each primer set, 5 µL of the DNA sample, and nuclease-free water, with the thermal protocol: 95 °C for 2 min, 35 cycles of 95 °C for 15 s, annealing temperature depended upon the specific primers (58–60 °C), 72 °C for 1 min, and 72 °C for 10 min. The PCR products were separated by 1.5% agarose gel electrophoresis (AppliChem, Darmstadt, Germany). A gene ruler 100 bp DNA ladder (Fermentas, Thermo, Offenbach, Germany) was used to determine the fragment size. The gel was photographed using a gel documentation system (Alpha Innotech, Biometra, South San Francisco, CA, USA)™, and size-specific DNA bands were excised and purified from gels using the QIAquick Gel Extraction Kit (Qiagen, Hilden, Germany). The purified products were used directly for cycle sequencing reactions using BigDye Terminator v3.1 Cycle Sequencing Kit (Applied Biosystems, Waltham, MA, USA). This was done by adding 2 BigDye 3.1 Sequencing Buffer, 1 μL BigDye Terminator, 3.2 pmol of the forward primer, and 3 μL of purified PCR product in a 10-μL reaction. The sequencing reaction was performed in 25 cycles of 96 °C for 15 s, 50 °C for 10 s, and 60 °C for 4 min. Reaction products were then purified using a Centrisep spin column (Applied Biosystems, Waltham, MA, USA) and sequenced on an ABI PRISM 3500 Genetic Analyzer (Life Technologies, Carlsbad, CA, USA). Thereafter, the obtained sequences were assembled and aligned using the Geneious Prime software, version 2021.1.1. https://www.geneious.com (accessed on 14 November 2021). BLAST® https://blast.ncbi.nlm.nih.gov/ (accessed on 14 November 2021) analysis of the obtained nucleotide sequences was performed to check sequence identities. In addition, identity matrices between chicken and human isolates were calculated and visualized with Geneious Prime software.

## 3. Results

### 3.1. E. coli Isolation, Identification, and Serotyping

Fifty-six farms were positive for *E.* *coli* isolation with a percentage of 46.7%. *E. coli* isolates were reported from internal organs (liver, lung, spleen, and heart) of 120 diseased broiler chickens that collected from farms located in Dakahlia, Giza, and Sharqia governorates; *E.* *coli* isolates were identified TSI acidic at slant and bottom with gas production, positive for catalase, methyl red, and indole, while negative for VP, oxidase, and citrate.

The chicken isolates were differentiated serologically and revealed different serotypes: O18 (*n* = 4), O55 (*n* = 8), O86a (*n* = 7), O111 (*n* = 6), O125 (*n* = 18), O127 (*n* = 5), O157 (*n* = 2), O159 (*n* = 4), and O166 (*n* = 2). On the other hand, all the nine human samples were positive for *E. coli* with the following different serotypes: O44 (*n* = 1), O55 (*n* = 3), O86a (*n* = 2), O164 (*n* = 2), and O119 (*n* = 1).

### 3.2. Antimicrobial Susceptibility Pattern of the Isolated E. coli

#### 3.2.1. Antimicrobial Sensitivity Test (AST)

A total of 56 chicken and 9 human *E. coli* isolates were analyzed. A similar trend was observed among the chicken and human isolates where the majority of the isolates were resistant to cephalexin, cephalothin, ampicillin, amoxicillin-clavulanic acid, sulfamethoxazole-trimethoprim, and cefotaxime, as shown in Table 2, with percentages of 100, 100, 92.9, 96.4, 82.1, and 76.8%, respectively, in chicken isolates and 100, 100, 100, 33.3, 55.6, and 55.6%, respectively, in human isolates. Detailed percentage of resistant, intermediate, and sensitive isolates to each antibiotic is outlined in Table 2 according to the CLSI standard.

#### 3.2.2. ESBL Screening Test

The disc diffusion method with four cephalosporins, to detect ESBL, showed that the highest rate of resistance was against cefotaxime (94.6%), followed by Aztreonam and ceftazidime (85.7%) and then Ceftriaxone (78.6%) for isolates obtained from chickens. Furthermore, *E. coli* isolated from the human samples showed resistance of Aztreonam and Cefotaxime (100%), Ceftazidime (88.9%), and Ceftriaxone (55.6%) (Table 3).

#### 3.2.3. Double Disc Synergy Test (DDST)

**Figure 1:**
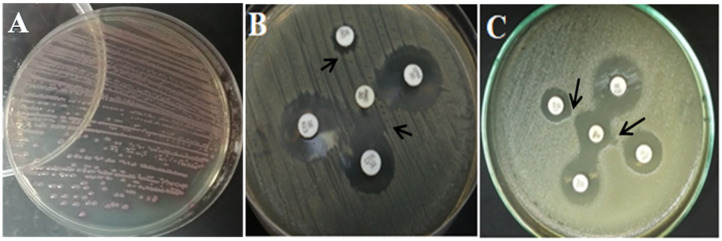
(**A**) *E. coli* on ESBL chromogenic agar. (**B**,**C**) Double Disc Synergy Test (DDST), which was done by using the DDST on four discs of cephalosporins antibiotic, i.e., cefotaxime, ceftriaxone, ceftazidime, and cefepime, against the amoxicillin–clavulanic acid. Black arrows show some synergistic pattern in the DDST.

DDST was used for the confirmation of ESBL production. The sample is considered positive when the inhibition zones around any of the cephalosporin discs are augmented in the direction of the disc containing clavulanic acid, which detected a different percentage for each antibiotic disc, as shown in Table 4 and Figure 1. ESBL production, in at least one of the antibiotics, was found in 42/56 (75%) of the chicken isolates and 8/9 (88.9%) of the human isolates.

Furthermore, all *E. coli* isolates from poultry and human were grown on the chromogenic agar, producing pink or purple colonies, indicating that all isolates were positive ESBL *E. coli* (resisting all applied antibiotics in this media, i.e., ceftazidime, cefotaxime, ceftriaxone, aztreonam, and fluconazole), as shown in Figure 1.

### 3.3. Molecular Detection and Identity Matrices

All screened ESBL genes were detected with a different ratio among the chicken isolates. The highest detected genes were *bla_CTX-M group9_* (*n* = 36/56) and *bla**_TEM_*** (*n* = 33/56). This was followed by *bla**_PER_*** (*n* = 27/56). The *bla**_OXA group 2_*** was found the lowest and was detected only once, as shown in Table 5.

Among the human *E. coli* isolates, only *bla_CTX-M group1_* and *bla_CTX-M group9_* were found in all isolates. The *bla**_GES_*** was found in 2 of 9. All remaining genes were not detected in any of the screened human isolates. Of note, the *bla_CTX-M group9_* showed the highest positive number in both chicken and human isolates.

**Figure 2:**
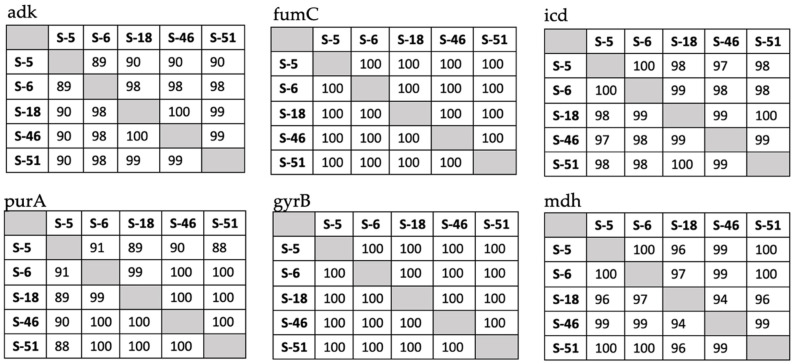
Nucleotide similarity between chicken samples (S18, S46, and S51) and human samples (S5 and S6).

Furthermore, nucleotide similarity was calculated between chicken and human isolates based on the sequence generated from the six housekeeping genes named *adk*, *fumC*, *gyrB*, *icd*, *mdh*, and *purA.* Identity of 100% has been found in *fumC* and *gyrB* among the chicken and human isolates. Interestingly, one human isolate (S-6) showed higher similarity for its *adk* and *purA* genes with the chickens isolates than the other human isolate. As shown in Figure 2.

## 4. Discussion

Unmanaged use of antibacterial drugs in human, veterinary, and even agricultural therapy has been proposed as a major cause for the selection and global spread of superbugs, including ESBL-variants [25,26]. Animal feed, particularly chicken feed, is highly supplemented with extended-spectrum antibiotics as prophylaxis/treatment or growth promotion. Generally, sub-therapeutic use of antibiotics in livestock including chicken may contribute to the global prevalence of resistant bugs such as ESBL-producing *E. coli* in the environment, posing a major health threat to human. Infections with ESBL-producing bacteria can be associated to treatment failure using common antibiotics, which may turn into a global health problem [2,7]. The risk for the transmission of ESBL-producing *E. coli* between chicken or chicken food products and humans has been highlighted in previous studies [11]; however, from Egypt, limited data on the spread between chickens and humans are available focusing only on either chickens or humans [17].

Here, we investigated the incidence and genetic relationship of ESBL-producing *E. coli* among chicken in three governorates in the northern part of Egypt. A total of 120 diseased chicken farms and an additionally nine fecal samples from diseased humans were subjected to primary bacteriological and biochemical analysis. In total, 46.7% of the examined chicken samples and 100% of the human samples tested positive for *E. coli*. Furthermore, serotyping analysis assigned all the isolates to O types, in line with Braun et al. [27], who demonstrated that ESBL-producing *E. coli* from Egyptian cattle are mainly O serotype [27]. Furthermore, ESBL-producers were detected using phenotypic characterization, reporting a high carriage of ESBL-producing *E. coli* in chicken isolates (75%). The reported high incidence of ESBL producers in chicken offal suggesting a potential contribution to the high detection rate of these superbugs in human fecal samples (88.9%). Since chicken-offal (liver, gizzard, and heart) is a popular fast food in Egypt and many developing countries as it is a cheap, easily prepared, and a good source of proteins [28]. The prevalence of ESBL-*E. coli* in chicken offal has been previously reported in Egypt and particularly in cities of northern Egypt. Studies in developing countries demonstrated ESBL-*E. coli* in chicken-offal [29,30]. In the other hand, lower incidence of ESBL-*E. coli* was described among diseased chickens (37.8), farm workers (37.8), and in the environment (24.3%) in Nigeria [31].

In addition to ESBL-production, the antibiotic resistance profile revealed that poultry and human isolates expressed a similar antibiotic resistance profile, including high resistance to ampicillin, amoxicillin-clavulanic acid, and sulfamethoxazole-trimethoprim. This is consistent with former reports that showed similar resistance pattern with ESBL-producing *E. coli* isolated from frozen chicken meat in Bangladesh [32] and broiler farms in the Philippines [33]. Moreover, studies in Japan and Korea recorded resistance of ESBL-producing *E. coli* associated with high resistance to ampicillin, amoxicillin, and sulfamethoxazole-trimethoprim [34,35]. The ESBL phenotypes were further confirmed using PCR, showing that among the genes responsible for ESBL resistance, *bla_CTX-M group9_*, and *bla_TEM_* were the highly predominant in chicken isolates followed with *bla**_PER_*** and *bla**_ctx-M group1_***. In great consistent with recent study in Japan that showed high prevalence rate of CTX-M and TEM families between ESBL-producing *Enterobacteria* isolated from domestic and farm animals [36]. Our analysis also revealed that only *bla_CTX-M group1_* and *bla_CTX-M group9_* were detected in human isolates and the individual data of chicken isolates revealed that the *bla_TEM_* and *bla_CTX-Mgroup9_* are common in Sharqia governorate, *bla_TEM_* and/or *bla_SHV_* with *bla_CTX-Mgroup1_* and/or *bla_CTX-Mgroup9_* are common in Dakahlia governorate, and *bla_TEM_* is a common gene present in Giza governorate. Moreover, a number of peer-reviewed articles, which included 1329 *E. coli* isolates from humans and various farm animals, including broilers, showed that ≥70% of the animal isolates and ≥50% of the human isolates widely harbor ESBL genes belonging to the CTX-M family, or the combinations of CTX-M + TEM or SHV + TEM families [37]. The ESBL resistance-associated genes *bla_TEM_*, *bla_SHV_*, and *bla_CMY_* were previously detected in *Enterobacteriaceae* isolates from healthy chickens [17] and heart blood from septicemic chickens in Egypt [38]. Another surveillance study also reported significant prevalence of *bla_CTX-M_* gene among ESBL-producing *E. coli* isolates from offal samples collected from 20 chicken farms distributed in four governorates in Egypt [39].

The bla*_CTX-M_*_group9_ variant greatly dominated in both chicken and human isolates. The CTX-M family is known with conferring resistance against to cefotaxime, one of the widely used antibiotics in veterinary medicine. This family was detected in livestock animals in Egypt and hospitalized patients in Alexandria, Egypt [27] and many food chain animals in different countries [40].

Furthermore, sequencing analysis revealed a significant degree of identity (>90%) among the ESBL-producing isolates from chicken as well as isolates retrieved from humans. This finding is similar to that obtained by Dahms et al. showing that ESBL-human isolate shared an identical CTX-M allele to the isolate found in the cattle fecal sample from the same farm [41]. Phenotypic and genotypic analysis indicates significant similarity between ESBL-producing *E. coli* from chicken and human, suggesting a potential transmission of these superbugs from chicken to human. Food of animal origin was recognized as the primary source of human colonization or infection with ESBL bugs [15,16,42]. Furthermore, a number of studies in many European countries reported contamination of chicken meat with ESBL-producing *E. coli* [16,43]. Globally, chickens are the most consumed animal, and contamination of these food sources with ESBL bugs may function as an effective disseminator of these bugs to humans. A systemic review study also attributed the high incidence of human colonization with ESBL-producing *E. coli* to direct or indirect contact with animals (via consumption) [44]. However, while our data suggested a potential transmission of ESBL-*E. coli* from chickens to humans, we admit that there are limitations in our study, as the chicken isolates were collected from the northern part of Egypt and human isolates from the southern Egypt. It may be difficult for a person from southern Egypt to have direct or indirect contact with chickens in northern Egypt, although it is not impossible, since many Egyptians from southern Egypt travel daily to northern Egypt, spend the day there, and then return south. Furthermore, northern Egypt has the highest number of large poultry enterprises compared to the other Egyptian cities [45]. Therefore, northern Egypt pumps frozen chicken and chicken products including chicken offal all over the country. Based on the nature of chicken products circulated throughout the country, the possible spread of infection among the population is not impossible.

## 5. Conclusions

The study shows the phenotypic and genetic links of ESBL-producing *E. coli* isolated from chickens and humans in Egypt, suggesting a possibility of transmission among chickens and humans. This highlights the need of continuous monitoring and obtaining more samples at a closer proximity “chicken-human interface”. In addition, deep genetic studies using whole genome sequencing is required for better understanding of the genetic relationship between animal and human isolates. Ultimately, a “One Health” approach should be more active in Egypt to avoid rapid evolution of antibiotic-resistance bacteria.

## Figures and Tables

**Table 1:** Specific primers for ESBL genes detection.

Gene	Sequence (5′–3′)	Temperatures of Annealing Step (°C)	Product Size	References
*bla_TeM-_*_F_ *bla_TeM-_*_R_	ATG AGT ATT CAA CAT TTC CGT TTA CCA ATG CTT AAT CAG TGA	58	861 bp	[23]
*bla_VeB-_*_F_ *bla_VeB-_*_R_	GCC AGA ATA GGA GTA GCA AT TGG ACT CTG CAA CAA ATA CG	58	703 bp	[9]
*bla_OXa2-_*_F_ *bla_OXa2-_*_R_	ATG GCA ATC CGA ATC TTC GC GCA CGA TTG CCT CCC TCT T	60	670 bp	[9]
*bla_OXa10-_*_F_ *bla_OXa10-_*_R_	ATG AAA ACA TTT GCC GCA TAT G TTA GCC ACC AAT GAT GCC CT	60	801 bp	[9]
*bla_ges-_*_F_ *bla_ges-_*_R_	TAC TGG CAG SGA TCG CTC AC TTG TCC GTG CTC AGG ATG AG	62	838 bp	[9]
*bla_PeR-_*_F_ *bla_PeR-_*_R_	CTC AGC GCA ATC CCC ACT GT TTG GGC TTA GGG CAG AAA GCT	62	851 bp	[9]
*bla_shV_*_-F_ *bla_shV_*_-R_	CGC CTG TGT ATT ATC TCC CTG TTA GCG TTG CCA GTG CTC GAT	64	849 bp	[9]
bla_CTX-M 1-F_ bla_CTX-M 1-R_	AGT TCA CGC TGA TGG CGA CG GAC GAT TTT AGC CGC CGA CG	67	839 bp	[9]
bla_CTX-M 9-F_ bla_CTX-M 9-R_	GCG TGC ATT CCG CTG CTG C ACA GCC CTT CGG CGA TGA TTC	67	832 bp	[9]

**Table 2:** Antimicrobial susceptibility pattern of the isolated *E. coli* from poultry and human.

Antimicrobial Agent	Resistant No. (%) ^1^		Intermediate No. (%) ^1^		Sensitive No. (%) ^1^	
	Poultry (*n* = 56)	Human (*n* = 9)	Poultry (*n* = 56)	Human (*n* = 9)	Poultry (*n* = 56)	Human (*n* = 9)
Amoxicillin-clavulanic acid (AMC^30^)	54 (96.4%)	3 (33.3%)	2 (3.6%)	1 (11.1%)	0	5 (55.6%)
Ampicillin (AMP^10^)	52 (92.9%)	9 (100%)	4 (7.1%)	0 (0%)	0	0 (0%)
Aztreonam (ATM^30^)	18 (32.1%)	2 (22.2%)	6 (10.7%)	2 (22.2%)	32 (57.2%)	5 (55.6%)
Cefepime (FEP^30^)	20 (35.7%)	3 (33.3%)	24 (42.9%)	3 (33.3%)	12 (21.4%)	3 (33.3%)
Cefotaxime (CTX^30^)	43 (76.8%)	5 (55.6%)	9 (16.1%)	2 (22.2%)	4 (7.1%)	2 (22.2%)
Ceftazidime (CAZ^30^)	30 (53.6%)	4 (44.4%)	16 (28.6%)	4 (44.4%)	10 (17.8%)	1 (11.1%)
Ceftriaxone (CRO^30^)	25 (44.7%)	3 (33.3%)	11 (19.6%)	0 (0%)	20 (35.7%)	6 (66.7%)
Cephalexin (CL^30^)	56 (100%)	9 (100%)	0 (0%)	0 (0%)	0 (0%)	0 (0%)
Cephalothin (KF^30^)	56 (100%)	9 (100%)	0 (0%)	0 (0%)	0 (0%)	0 (0%)
Ciprofloxacin (CIP^5^)	37 (66.1%)	1 (11.1%)	7 (12.5%)	0 (0%)	12 (21.4%)	8 (88.9%)
Colistin sulphate (CT^10^)	23 (41.1%)	0 (0%)	0 (0%)	0 (0%)	33 (58.9%)	9 (100%)
Imipenem (IPM^10^)	0 (0%)	0 (0%)	0 (0%)	0 (0%)	56 (100%)	9 (100%)
Norfloxacin (NOR^10^)	37 (66.1%)	1 (11.1%)	4 (7.1%)	0 (0%)	15 (26.8%)	8 (88.9%)
Sulfamethoxazole-trimethoprim (SXT^25^)	46 (82.1%)	5 (55.6%)	1 (1.8%)	0 (0%)	9 (16.1%)	4 (44.4%)

^1^ Percentage of positive samples.

**Table 3:** ESBL screening test using the disc diffusion method for isolates obtained from poultry and humans.

Antibiotic Disc for ESBL Screening Test	Interpretation of Conduct ESBL-Testing	ESBL Production Screening	
		Poultry (%) ^1^	Human (%) ^1^
Aztreonam (ATM^30^)	≤27 mm	48 (85.7%)	9 (100%)
Cefotaxime (CTX^30^)	≤27 mm	53 (94.6%)	9 (100%)
Ceftazidime (CAZ^30^)	≤22 mm	48 (85.7%)	8 (88.9%)
Ceftriaxone (CRO^30^)	≤25 mm	44 (78.6%)	5 (55.6%)

**^1^** Percentage of positive samples from a total of 56 (chickens) and 9 (humans) tested samples.

**Table 4:** ESBL disc confirmation by the Double Disc SynergyTest (DDST). The confirmation was considered positive if the cephalosporin inhibition zone was extended toward the clavulanic acid antibiotic.

Antibiotic Disc for ESBL Screening Test	ESBL Production Confirmation	
	Poultry (%) ^1^	Human (%) ^1^
Cefepime (CPM^30^)	22 (39.3%)	8 (88.9%)
Cefotaxime (CTX^30^)	22 (39.3%)	7 (77.8%)
Ceftazidime (CAZ^30^)	20 (35.7%)	6 (66.7%)
Ceftriaxone (CRO^30^)	28 (50%)	8 (88.9%)

**^1^** Percentage of positive samples from a total of 56 and 9 tested samples obtained from chickens and humans, respectively.

**Table 5:** PCR results of chicken (56) and human (9) samples for detection resistance-associated genes.

Antibiotic Resistance Genes	PCR Result: Positive Result/Total Examined Isolates (%)	
	Poultry	Human
*bla_TEM_*	33/56 (58.9%)	0/9 (0%)
*bla_VEB_*	7/56 (12.5%)	0/9 (0%)
*bla_OXA group 2_*	1/56 (1.8%)	0/9 (0%)
*bla_OXA group 10_*	9/56 (16.1%)	0/9 (0%)
*bla_GES_*	10/56 (17.9%)	2/9 (22.2%)
*bla_PER_*	27/56 (48.2%)	0/9 (0%)
*bla_SHV_*	14/56 (25%)	0/9 (0%)
*bla_CTX-M group 1_*	19/56 (33.9%)	9/9 (100%)
*bla_CTX-M group 9_*	36/56 (65.3%)	9/9 (100%)

## Data Availability

No supporting data was included.
